# Drought exposure leads to rapid acquisition and inheritance of herbicide resistance in the weed *Alopecurus myosuroides*

**DOI:** 10.1002/ece3.8563

**Published:** 2022-02-16

**Authors:** Vian H. Mohammad, Colin P. Osborne, Robert P. Freckleton

**Affiliations:** ^1^ Department of Animal & Plant Sciences University of Sheffield Sheffield UK

**Keywords:** *Alopecurus myosuroides*, blackgrass, epigenetics, genetic inheritance, physiological pathway, stress response

## Abstract

Globally, herbicide resistance in weeds poses a threat to food security. Resistance evolves rapidly through the co‐option of a suite of physiological mechanisms that evolved to allow plants to survive environmental stress. Consequently, we hypothesize that stress tolerance and herbicide resistance are functionally linked. We address two questions: (i) does exposure to stress in a parental generation promote the evolution of resistance in the offspring? (ii) Is such evolution mediated through non‐genetic mechanisms? We exposed individuals of a grass weed to drought, and tested whether this resulted in herbicide resistance in the first generation. In terms of both survival and dry mass, we find enhanced resistance to herbicide in the offspring of parents that had been exposed to drought. Our results suggest that exposure of weeds to drought can confer herbicide resistance in subsequent generations, and that the mechanism conferring heritability of herbicide resistance is non‐genetic.

## Graphical abstract

We show that exposure to drought stress in parents leads to enhanced herbicide resistance in their offspring. This is inherited non‐genetically.

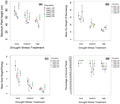

## 1 INTRODUCTION

Since the origins of agriculture, arable weeds have been among the most important biotic factors limiting crop production (Matzrafi et al., 2014; Oerke, 2006). Weeds cause yield reductions of up to 34% across the globe, and are thereby a significant threat to food security (Matzrafi et al., 2014; Oerke, 2006). Resistance to herbicides is a key driver of these losses, and currently around 220 herbicide‐resistant weed species have been confirmed throughout the world (Heap, 2014). There are major unanswered questions, however, such as why some species regularly evolve resistance and others do not, or what ecological factors predispose some species to rapidly evolve resistance.

Plants are continuously exposed to a range of biotic and abiotic factors that reduce growth, productivity, and reproductive success (Kinoshita & Seki, 2014). To survive, plants have, therefore, evolved a range of physiological mechanisms and strategies to survive a range of external pressures, such as drought, heat, cold, and physical damage (Anjum et al., 2011; Boyko & Kovalchuk, 2008; Ding et al., 2012; Fu & Dong, 2013; Goh et al., 2003; Golldack et al., 2011; Kinoshita & Seki, 2014; Wang et al., 2011). Resistance to herbicide is an important example of rapid evolution (Moss et al., 2019): when weeds within crop fields are repeatedly exposed to herbicides with the same mechanism of action, selection for herbicide resistance occurs (Jugulam & Shyam, 2019). Herbicide resistance is the ability of weed plants to survive following a herbicide treatment that would usually be expected to be lethal to the wild type (Katerova & Miteva, 2010; Reade et al., 2004). Herbicide resistance in weeds is conferred by one of two broad mechanisms: monogenic target‐site resistance (TSR) or non‐target‐site resistance (NTSR). Of the two forms of resistance, NTSR mechanisms of herbicide resistance appear to be more common than target site mechanisms (Delye et al., 2013; Ge et al., 2010; Jugulam & Shyam, 2019; Shaner et al., 2012).

Non‐target‐site resistance is mediated by a subset of physiological pathways responsible for responses to abiotic stress, many of which are also induced in weeds by herbicide application (Cummins et al., 1997; Delye, 2013; Letouze & Gasquez, 2001). Plants have evolved complex physiological systems of stress detection, response, and signaling that activate both specific and general responses (Vaahtera & Brosche, 2011). The physiological basis of NTSR is usually the stimulation of herbicide metabolism or detoxification mediated by cytochrome P450 monooxygenases (CYPS) (Vila‐Aiub et al., 2009), glutathione S‐transferases (GSTs) (Reade et al., 2004), and other Phase II metabolism enzymes (Powles & Yu, 2010).

Among these various routes, pathways that remove reactive oxygen species (ROS) are particularly important. Mechanisms of detoxification have been reported in driving the herbicide resistance of major weed species (Delye et al., 2013; Kreuz et al., 1996). For example, GSTs play major roles in oxidative stress metabolism, although the mechanisms of their regulation are not well understood (Chen et al., 2012). In metabolizing herbicides, these mechanisms cause a decrease in the amount of herbicide that reaches its target site, thereby preventing lethal herbicide action (Cummins et al., 1997; Letouze & Gasquez, 2001; Yuan et al., 2007). Glutathione S‐transferases have also been linked with responses to biotic and abiotic stress (Frova, 2006; Moons, 2005). NTSR mechanisms can be affected by alterations in environmental conditions (Matzrafi, 2019). Under different environmental conditions, both biotypes (herbicide‐resistant and susceptible) have shown increased and decreased tolerance to herbicides (Jugulam & Shyam, 2019). Altering environmental conditions can, therefore, impact herbicide effectiveness and favor the selection of further tolerant biotypes. Therefore, knowledge of how NTSR mechanisms behave in changing environmental conditions is essential (Busi et al., 2013; Jugulam & Shyam, 2019).

Plants have the ability to ‘remember’ previous stress exposure, and can benefit from this when re‐exposed in the future (Kinoshita & Seki, 2014; Onate et al., 2011; Tahkokorpi et al., 2007a, 2007b). Ding et al. (2012) in a study on *Arabidopsis* plants found that following exposure to drought stress conditions, plants respond to subsequent stress by increased rapid adaptive gene expression, compared with plants not previously exposed to a drought stress. This phenomenon has been termed the “priming effect” (Tanou et al., 2012) or “stress memory” (Ding et al., 2012; Miryeganeh, 2021; Walter et al., 2013). A series of mechanisms are assumed to be involved in responses of plants to prior stress exposure (Scholes & Paige, 2015), including physiological, metabolic, and morphological changes (Bruce et al., 2007; Walter et al., 2013).

In addition to mechanisms that prime individual plants, inter‐generational stress memory is also possible. Epigenetic mechanisms are thought to play an essential role in the regulation of the expression of stress response genes (Chinnusamy & Zhu, 2009; Miryeganeh, 2021), via small RNAs, histone modifications, and DNA methylation. These can be passed on to the next generation, generating inter‐generational stress ‘memory’ (Chinnusamy & Zhu, 2009; Kinoshita & Seki, 2014; Miryeganeh, 2021). Epigenetic mechanisms have been shown to regulate genetic functions, such as replication, transcription, DNA repair, gene transposition, and cell differentiation. Both the generation of small RNAs and modifications in chromatin have been shown to contribute to transcriptional and post‐transcriptional control of gene expression, which is crucial for environmental stress responses (Angers et al., 2010; Madlung & Comai, 2004; Miryeganeh, 2021). The role of such effects in the inheritance of herbicide resistance is, however, largely unexplored.

Currently, the most significant weed in Northern Europe is blackgrass (*Alopecurus myosuroides*), with recent increases in this species being correlated directly with herbicide resistance (Hicks et al., 2018). In the United Kingdom, the loss of weed control resulting from herbicide resistance incurs an economic cost of approximately 0.5 bn GBP per year in wheat production, associated with one million ton per year of yield loss (Varah et al., 2020). In common with other grass weeds, *A*. *myosuroides* is an obligate out‐crosser with a self‐incompatible reproduction system (Chauvel & Gasquez, 1994). This type of reproduction has the ability to enhance the spread of herbicide resistance in the weed population (Matzrafi et al., 2014). Currently *A*. *myosuroides* is distributed widely in the UK, with its distribution being linked with heavy and wet soils both at field (Metcalfe et al., 2016, 2018) and national scales (Hicks et al., 2021). Both NTSR and TSR mechanisms confer resistance of *A*.*myosuroides* to a range of herbicides, but NTSR is generally more common (Comont et al., 2020; Hicks et al., 2021).

In this paper, we explore the link between stress exposure and the evolution of herbicide resistance in grass weed *A*. *myosuroides*. The first major question we address is whether exposure to stress leads to the evolution of herbicide resistance in the subsequent generation of droughted parental plants. The second question is whether such rapid evolution of herbicide resistance is heritable through an epigenetic mechanism? Our results show that stress exposure can induce herbicide resistance in a subsequent generation, and that this is inherited non‐genetically.

## 2 MATERIALS AND METHODS

To investigate whether exposure to stress can stimulate the evolution of herbicide resistance in a subsequent generation of *A*. *myosuroides*, we carried out two experiments. The first experiment investigated whether herbicide resistance occurs in first‐generation (F1) offspring of droughted parent plants. The second experiment was designed to investigate the possible roles of non‐genetic mechanisms in inheritance of herbicide resistance in *A*. *myosuroides*.

### 2.1 Experiment 1: Investigation of rapid evolution of herbicide resistance following drought

Seeds of five populations of *A*. *myosuroides* were obtained from Herbiseed, Ltd, UK. The seeds had no previous treatments of herbicides and are regarded as research industry susceptible (All the information regarding the seeds was obtained at: www.herbiseed.com, in section “Full documentation of experimental population for GLP & GEP”), however, the source is not available. As described below, when exposed to herbicides, plants grown from these seeds showed very mortality as would be expected of susceptible populations.

In March 2015, nine seeds of each of the five populations were planted in square plastic pots (200 mm). In this and subsequent growth trials, a standard potting mixture 1:1 was used (50% peat free compost + 50% vermiculite) to a planting depth of 50 mm, with a saucer placed underneath each pot to avoid losing nutrients by leaching. Pots were maintained in a greenhouse with average day and night temperatures of 20 and 15°C, respectively, and well‐watered to ensure seed germination. Following emergence, seedlings were thinned to three plants per pot (a similar in height of ~40 mm, and number of leaves, 1 leaf) to ensure sufficient plant material. All the information on planting dates and other measurements are summarized in Figures S1 and S2. Plant height, aboveground biomass, and seed weight were measured to estimate the influence of drought treatments on the phenotype. In addition, surviving and dead plants were assessed to evaluate the tolerance of *A*. *myosuroides* to drought stress.

The drought treatments were initiated 30 days after emergence. A low drought treatment was applied by withholding water until the shoots of approximately 25% of plants had died back. The first period of low drought treatment was started on 10th April, 2015 until 12th May, 2015 (roughly 35 days); the second period was started on 18th May, 2015 until 12th June, 2015 (21 days); and the final period of drought treatment was applied on 18th June, 2015 until 22nd July 2015 (35 days). A high drought treatment was applied by withholding water until 75% of plants had died back. The first period of high drought treatment was started on 10th April, 2015 until 21st May 2015 (approximately 42 days); the second period was applied on 27th May, 2015 till 23rd June, 2015 (roughly 28 days); and the last period was initiated on 29th June, 2015 until 30th July, 2015 (28 days). Following each period of drought treatment, the plants were re‐watered as normal watering (twice per week) until the appearance of shoots. Visual assessment by the same observer was made to monitor the growth and mortality rate of each. In addition, the soil moisture content of each pot was monitored after each period of drought by measuring the apparent dielectric constant (ThetaProbe, Delta‐T‐Devices, Cambridge‐England).

The experiment was conducted as a randomized block design with four replicates of the three treatments. Plant height was recorded 48 days after germination from May to August 2015, at the end of each drought period and before re‐watering the plants. The shoots of all plants were measured at soil level to the end of the longest leaf. During the anthesis stage and before pollen emission, the plants were covered by a pollen‐proof bag to ensure cross‐pollination with members of the same population only (Neve & Powles, 2005).

During harvest, the aboveground biomass of a single mature plant per pot was harvested (2nd September–9th September, 2015 for the high drought treatment). This allowed the impact of water deficits on aboveground biomass production to be evaluated. Aboveground biomass was hand‐harvested and directly weighed with a scale (EP 6102C, max 100 g, *d* = 0.01 g, Ohaus Corporation, Parsippany, NJ, USA). Following harvest, seeds of each plant were separated and weighed using a high precision scale (GH‐252‐EC, max = 250 g, min = 1 mg, *d* = 0.01/0.1 mg, A&D Instruments, Abingdon, UK). Seeds were stored in dark and dry conditions (fridge 4°C) until further use.

#### 2.1.1 Herbicide assay

Nine seeds from the F1 of all populations were planted in circular plastic pots (100 mm in diameter, 215 mm depth, and 4 L capacity). Following sowing, pots were thoroughly watered from above to ensure germination and, through the course of the experiment, plants were watered as required. Following emergence, seedlings similar in height and number of leaves (height: 40 mm, and 1 leaf) were thinned to three plants per pot. At the 2–3 tiller stage, these seedlings were sprayed with fenoxaprop‐P‐ethyl herbicide (as “Puma Super” – 69 g a.i./L, Bayer Crop Science) using two different doses, a lethal dose (40 g a.i./h) and sub‐lethal dose (20 g a.i./h). We used fenoxaprop‐P‐ethyl herbicide because resistance to fenoxaprop‐P‐ethyl is linked with the selection for NTSR (Delye et al., 2007, 2015; Letouze & Gasquez, 2001). Herbicides were applied using a flat nozzle sprayer (3l capacity) delivering 0.79 gallons 0.20 min^−1^ (equivalent to 4 gallons/min with pressure up to 100 PSI) herbicide in Max 45 PSI, applied with a fine spray and 3BAR pressure.

Twenty‐eight days after herbicide application, dead and damaged plants were assessed. Plants were scored as damaged if they had yellow or burned leaves following herbicide treatment. Surviving plants were categorized in two ways to account for the differential outcomes of exposure to herbicide: plants were categorized as ‘surviving’ if they showed no visible effects of herbicide exposure, or ‘damaged’ if they survived but with obvious effects on aboveground tissues. To evaluate the impacts of herbicide exposure, we first combined surviving and damaged individuals, and calculated these as a proportion of the plants treated. Second, we calculated the proportion of plants which survived, compared with the fraction of those that died or were damaged. These two approaches measure resistance in slightly different ways. The first measures the plants that survive application, whether they are damaged or not, while the second measures those plants that are unaffected by the herbicide application. The dry weight of each surviving and damaged plant was measured using high precision scales (GH‐252‐EC, A&D Instruments).

The herbicide assay was conducted as a randomized block design: there were 30 pots per block (five blocks in total), five populations of *A*. *myosuroides*, two levels of herbicide doses, and as pre‐treatment three levels of drought stress for a total of 150 pots.

### 2.2 Experiment 2. The role of non‐genetic inheritance

In July 2016, seeds of *A*. *myosuroides* were collected from 15 arable winter cereal fields across England. Following collection, seeds were threshed and cleaned to eliminate unfilled seeds and debris, then stored in a paper bag in dark/dry condition (i.e., fridge 4°C) until needed. In October 2016, the seeds of all populations were located in an incubator at 30°C for 42 days to break primary seed dormancy.

#### 2.2.1 Plant cloning and drought stress treatment

In March 2017, nine seeds of each population were sown at a planting depth of 50 mm below the soil surface. The pots were maintained in a greenhouse with a 14‐h day length and supplementary lighting. Temperature was set to 23°C during daylight hours, and 15°C during nighttime. After sowing, pots were well‐watered thoroughly from above to ensure seed germination. Following emergence, seedlings similar in height ~40 mm and number of leaves (1 leaf) were thinned to three plants per pot to ensure sufficient plant material.

In April 2017, 35 days after sowing (3–4 tiller stage), each plant was divided into two clones. Plants were cloned to produce two identical seedlings for the investigation of the role of epigenetic mechanism in herbicide resistance evolution. The root of each cloned plant was cut to approximately 1 cm, and the plant shoots were trimmed 4‒5 cm. The cloned plants were replanted in a clone‐propagation tray for 2 weeks. On April 24th 2017, all the cloned plants were re‐potted in plastic pots same size and mixture contain as previously described, and allowed to establish for 1 week before initiating a drought stress treatment.

A high drought treatment was applied to half of the pots by withholding water until the shoots of approximately 75% of plants had died back. The first period of high drought treatment was started on 1st May, 2017 until 23rd May, 2017 (approximately 21 days); the second period was started on 28th May, 2017 until 22nd June, 2017 (roughly 28 days); and the final period of drought treatment was applied on 28th June, 2017 until 30th July 2017 (more than 27 days). After each period of drought treatment, the plants were re‐watered as normal watering (twice per week) until the appearance of shoots. The experiment was conducted as randomized complete‐block design with six replicates (blocks).

Plant height, aboveground biomass, and seed weight were measured, in addition to the number of surviving and dead plants. Plant height was recorded before the harvest of plants in August, 2017. The shoots of all plants were measured from the soil level to the end of the longest flowering shoot. During the anthesis stage and before pollen emission, the *A*. *myosuroides* plants were covered (three pots together) by a pollen‐proof bag to ensure that cross‐pollination only occurred among members of the same population (Neve & Powles, 2005).

At harvest time, following 42 days of withholding irrigation, the aboveground biomass of a single mature plant per pot was harvested 15th September, 2017. This allowed the impact of watering treatments on aboveground biomass production to be evaluated. Aboveground biomass was hand‐harvested and directly weighed with a scale (EP 6102C, max 100 g, *d* = 0.01 g, Ohaus Corporation, Parsippany, NJ, USA). Following harvesting, seeds of each plant were separated and weighed using a high precision scale (GH‐252‐EC, max = 250 g, min = 1 mg, *d* = 0.01/0.1 mg, A&D Instruments, Abingdon, UK). Seeds were stored in dark and dry conditions (4°C) until further use. Plant height, biomass, and seed production were measured.

#### 2.2.2 Response to herbicide exposure

The seeds of all populations were placed in an incubator at 30°C for 42 days to break primary seed dormancy. The herbicide assay was carried out using the five populations that possessed a high rate of viability and germination rate in both treatments (“none” and “high” drought). Nine seeds of the F1 generation of the five populations were planted. Temperature was set to 23°C during daylight hours, and 15°C during night time with a 14‐h day length and supplementary lighting. Through the course of the experiment, plants were watered as required. Following emergence, seedlings similar in height and number of leaves (height: 40 mm, and 1 leaf) were thinned to three plants per pot.

At the 3–4 leaves stage in September 2018, the seedlings were sprayed with fenoxaprop‐p‐ethyl herbicide (“Puma Super” – 69 g a.i./L, Bayer Crop Science) using two different doses as previously described. There were four replicate pots per population for both drought treatments by dose combination, and there were two pots per dose per drought treatment with the five *A*. *myosuroides* populations. There were 20 pots per block (10 blocks in total, giving 200 pots) and the pots were completely randomized within blocks.

Twenty‐eight days after herbicide application (October 2018), dead and damaged plants were assessed as described above. Plants were grown to maturity in the greenhouse condition, to allow production of seed. During the flowering stage and before pollen emission, plants were covered (same treatment within same population pots together) by a pollen‐proof bag to ensure that cross‐pollination only occurred among members of the same population (Neve & Powles, 2005). In January 2019, the shoots of all plants were measured from the soil level to the end of the longest flowered shoot, then biomass and seed weight recorded as above.

### 2.3 Statistical analysis

#### 2.3.1 Analysis of Experiment 1

Linear Models and Generalized Linear Models were used to analyze the response of the parental plants (P) generation to drought treatment. Population and replicate were entered into the model as a factors in linear models. Plant height, biomass, and seed mass were log transformed. Generalized Linear Model (glm) with binomial errors was used to analyze the survival plants of drought treatments.

To explore the effect of drought stress in P generation on herbicide resistance in the F1 generation, Generalized Linear Models with a binomial error were used to analyze survival. The main hypothesis was that exposure of the P generation to drought would influence the response of the F1 generation to herbicide exposure. In the models for the F1 generation, an interaction between the P generation exposure to drought and herbicide treatment was, therefore, included.

#### 2.3.2 Analysis of Experiment 2 (epigenetic experiment)

Linear mixed effects analysis of the response of the cloned parental (P) generation to drought treatment was performed. To test the effects on plant height, biomass, and seed production, a Gaussian error distribution was assumed. Drought was entered into the model as a fixed effect. Clone ID was included as a random effect. Data on the effect of drought in cloned plants on plant height, biomass, seed mass, and the survivorship were analyzed.

Generalized Linear Mixed Models were used to analyze the effect of drought stress in parental cloned plants upon the survival of plants following herbicide application in the F1 generation. We again assumed a binomial error structure in this experiment as our dependent variable was a binomial outcome. We entered herbicide and drought (with an interaction term) as fixed effects in the model. Blocks and clone ID were entered as random effects. All analyses were conducted in R (R Core Team, 2016).

## 3 RESULTS

### 3.1 Herbicide resistance in offspring of droughted parental plants

**FIGURE 1:**
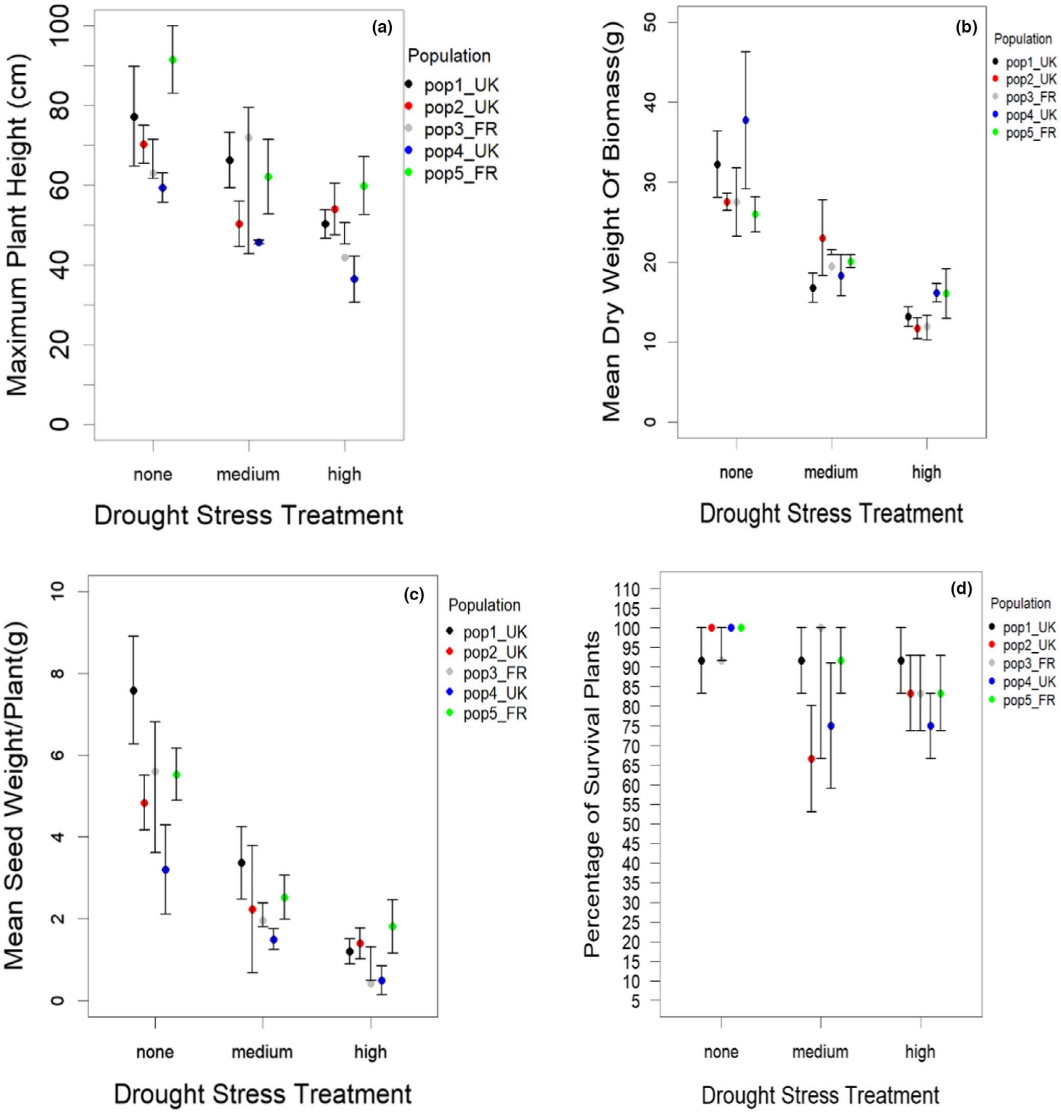
Effect of drought stress levels on *A*. *myosuroides* (a–d); maximum plant height (a), dry weight of aboveground biomass (b), seed production per plant (c), and percentage of survival of plants (d) across all the populations (Pop1–Pop5)

The drought treatments significantly affected plant height [Figure 1a, Table S1; *F*_2, 50_ = 14.8, *p *< .001], biomass [Figure 1b, Table S1; *F*_2, 50_ = 42.1, *p *< .001], and seed weight [Figure1c, Table S1; *F*_2, 49_ = 33.9, *p* < .001] of *A*. *myosuroides*. The range of survival for plants in the medium drought treatment was >65% to 100%, while the range in the high drought treatment was >75% to 95% (Figure 1d), indicating marginal effects of the drought treatments on survival. Therefore, although large numbers of the plants died back completely during the drought treatments, almost all of them regrew upon re‐watering. These results confirm that the drought treatment significantly impacted plant performance, relative to controls.

**FIGURE 2:**
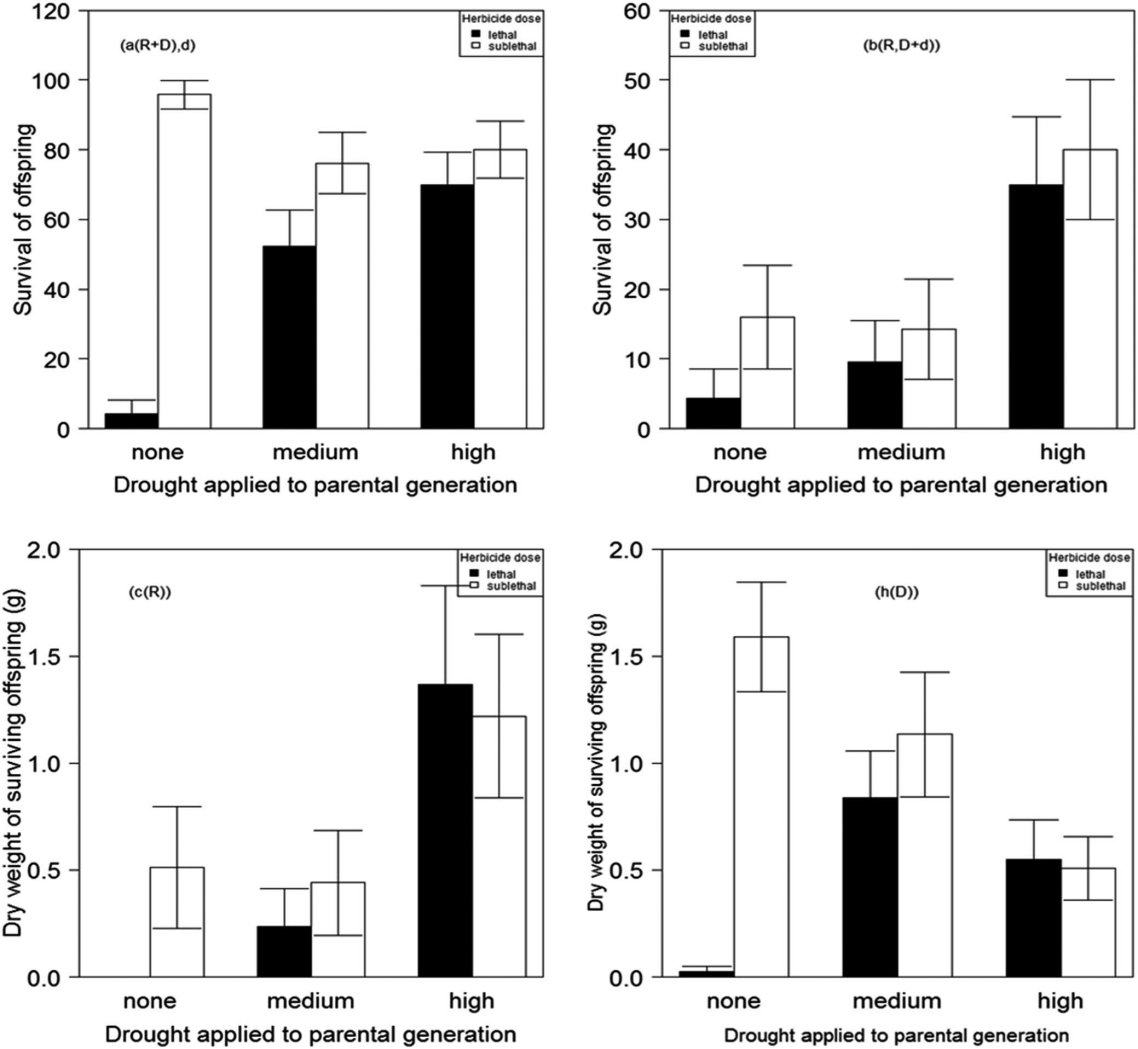
The effect of drought stress levels on the response of five *A*. *myosuroides* populations treated with lethal and sub‐lethal doses of fenoxaprop‐p‐ethyl herbicide. (a) survival of offspring when the resistant and damaged plants were combined. (b) survival of offspring when the damaged and dead plants were combined. (c and h) represent the effect of fenoxaprop‐p‐ethyl on the dry weight of intact and damaged plants, respectively

There were significant effects of herbicide treatment (χ^2^ = 31.30, df = 1, *p* < .001) and the previous exposure of the P generation to drought treatments (χ^2^ = 7.86, df = 2, *p* < .05) on the fraction of the F1 generation surviving following exposure to herbicides, measured as the number of plants surviving apparently intact (Figure 2a, Table S2a). However, this result was not significant when surviving plants were measured as intact plants versus damaged and dead plants (Table S2b: χ^2^ = 1.02, *df* = 1, *p* > .1).

There was significant interaction between previous drought exposure and herbicide treatment (χ^2^ = 28.36, *df* = 2, *p* < .001) in terms of dry weight, the performance of surviving plants (dead and damaged) mirrored the outcome with respect to survival. There was a significant interaction between herbicide application and exposure of the parental generation to drought (Figure 2c, Table S3: *F*_2, 67_ = 5.20, *p* = .01).

### 3.2 Non‐genetic resistance to herbicide by offspring of droughted plants

**FIGURE 3:**
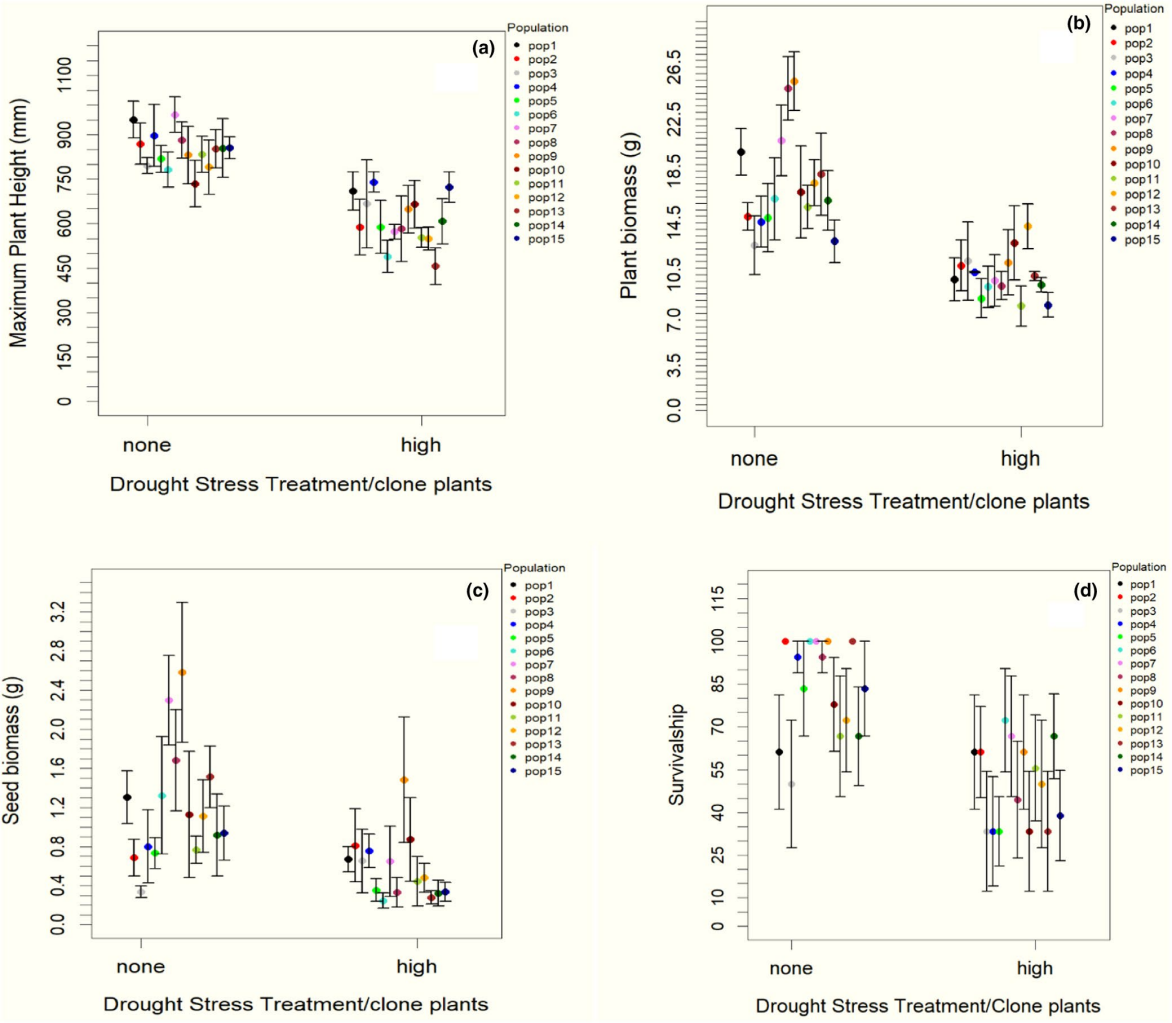
Effect of drought stress on cloned parent populations of *A. myosuroides*: maximum plant height (a), dryweight of aboveground biomass (b), seed production per plant (c), and the percentage of survival of plant (d)

As in the previous experiment, drought stress significantly affected plant height, biomass, and seed weight, confirming the impact of the treatment. Plant height is lower in high drought treatment, by about 0.47 mm ± 0.06, *p* < .001 (Figure 3a, Table S4A) and similarly plant biomass was lower by 0.70 g ± 0.1, *p* < .001 (Figure 3b, Table S4B). In addition, significant reductions were observed in seed weight −1.55 g ± 0.21, *p* < .001 (Figure 3c, Table S4C). Exposure to the drought treatment resulted in increased mortality in each population −2.27 ± 0.28, *p* < .001 (Figure 3d, Table S4).

**FIGURE 4:**
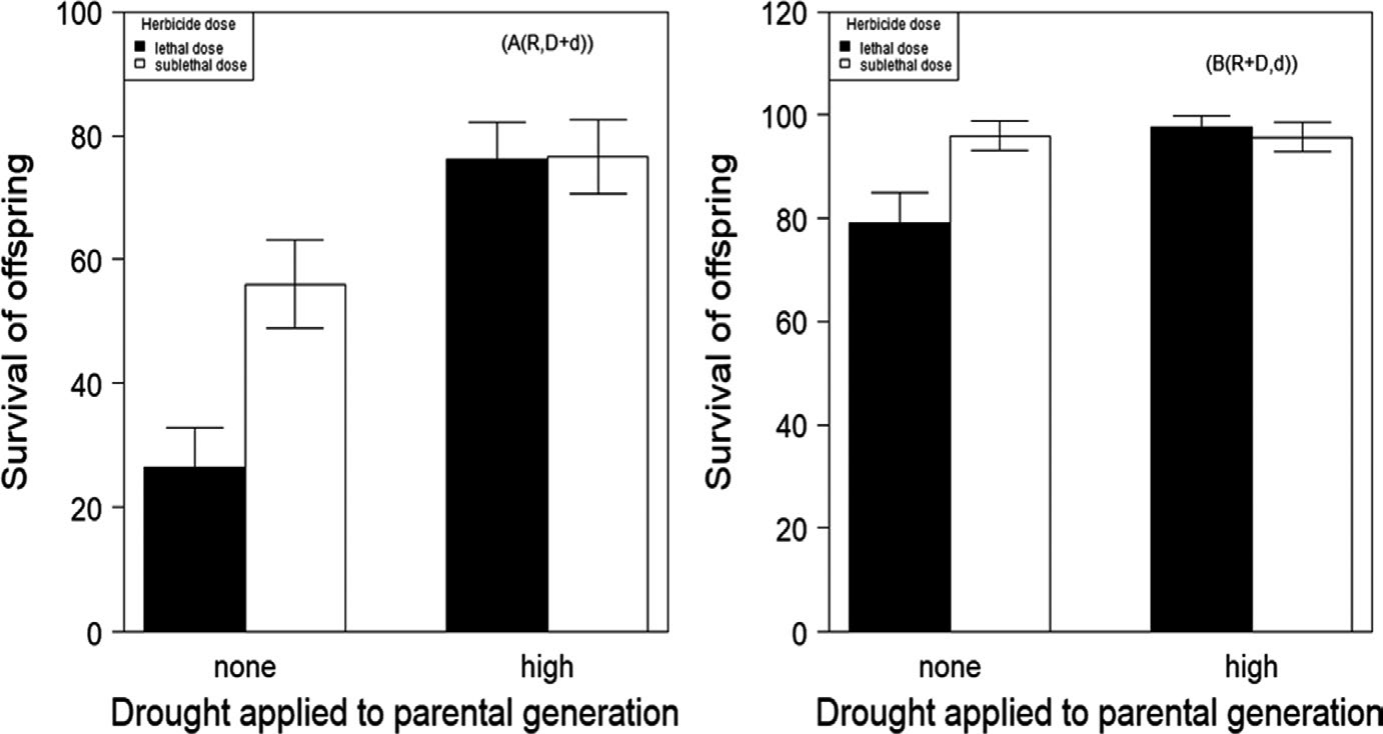
The effect of high drought stress on the survival cloned parental populations of *A*. *myosuroides* (a, b); populations treated with lethal (40 g a. i.) and sub‐lethal (20 g a. i.) doses of fenoxaprop‐p‐ethyl herbicide. (a(R, D+d)): survival of offspring when the damaged and dead plants were combined. (b(R+D, d): survival of offspring when the resistant and damaged plants were combined. Error bars are ± one standard error of the mean

**FIGURE 5:**
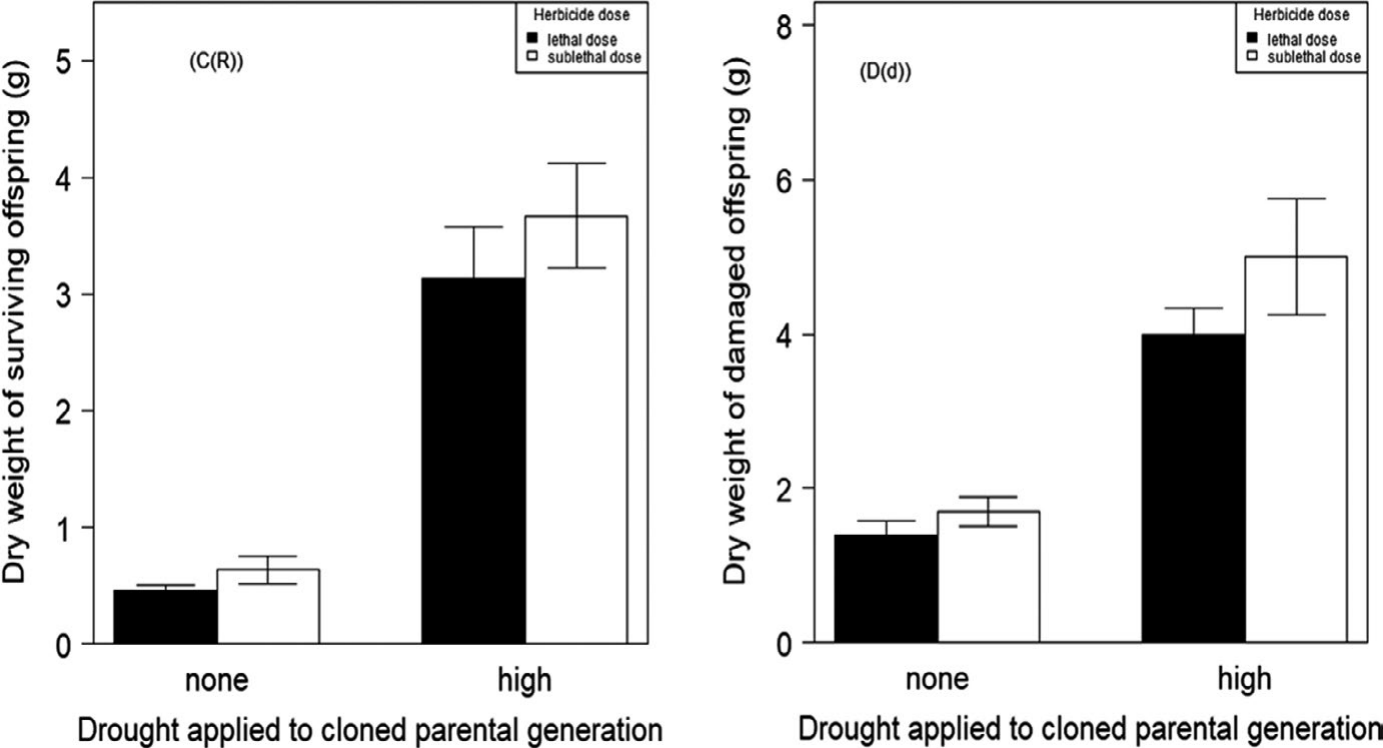
Effect of high drought stress in parental generation and herbicide treatment (lethal (40 g a. i.) and sub‐lethal (20 g a. i.) doses of fenoxaprop‐p‐ethyl herbicide on dry weight of resistant (c (R)) and damaged plants (d (d)) in first generation (F1) of *A. myosuroides*)

The application of herbicides differentially affected plants depending on whether the parent clones had been exposed to drought or not (Figure 4; Table S6). Among offspring of plants that had been exposed to no drought, there was much lower survival, with the proportion of plants damaged by the herbicide application being much greater (comparing proportions in Figure 4a,b; Table S6). Note, although the experiment did not include a zero herbicide application, the damage recorded (i.e., chlorosis and death of leaves) are direct consequences of herbicide application: these impacts were dramatically greater in the clones of parents not exposed to drought (Figures 4 and 5).

Drought applied to the P generation generated herbicide resistance in the F1: herbicide treatment significantly affected the occurrence of herbicide resistance in F1 plants from parents that experienced high drought (3.67 ± 0.45, *p* < .001) in comparison with well‐watered plants. This was true for the number of plants surviving apparently intact versus those that were damaged plus dead (Figure 5a, Table S5A). Additionally, when resistance was measured as plants that survived either damaged or intact together versus those that were dead (Figure 5b, Table S5B) in offspring of high droughted plants following the herbicide treatment there was also a strong effect (2.12 ± 0.48, *p* < .001). Furthermore, there was a significant interaction between drought applied to the P generation and herbicide application in the F1 (−1.86 ± 0.58, *p* < .001) in both combinations (resistant vs. damaged + dead) and (resistant + damaged vs. dead). This is an indication of a significant impact of drought stress exposure upon the evolution of herbicide resistance in the F1 generation.

We analyzed the dry weight of resistant plants, that is, the dry weight of F1 plants that survived the herbicide application. We constructed a linear model of resistant dry weight as a function of clone ID, herbicide, and drought. Statistically there was no significant effect of herbicide application on dry weight of resistant F1 plants (Figure 5, Table S7: *F*_1,103_ = 0.30, *p* > .1), while drought treatment as a factor had a significant effect on the dry weight of resistant plants (*F*_1,103_ = 81.27, *p *< .001). There was no significant interaction between herbicide application and exposure of P generation to drought for *A*. *myosuroides*. To analyze the dry weight of damaged plants, we performed a linear mixed effects analysis and there was no significant difference between sub‐lethal and lethal levels of herbicide application on the dry weight of damaged plants in F1 generation, but drought treatment of the parents had a significant impact on the dry weight of the offspring as one of the fixed effects 1.36 ± 0.39, *p* < .001 (Table S8).

## 4 DISCUSSION

Globally, weed herbicide resistance is growing rapidly (Heap, 2014). Despite the fact that TSR can endow high levels of herbicide resistance (Powles & Yu, 2010; Preston et al., 2009), the current rapid increase in the frequency of herbicide resistance is thought to be mainly due to NTSR (Delye et al., 2013; Ge et al., 2010; Shaner et al., 2012). It is important, therefore, to understand the genetic basis and eco‐evolutionary mechanisms underpinning the emergence of herbicide resistance. This study suggests a close relationship between response to abiotic stress and the rapid acquisition of herbicide resistance in *Alopecurus myosuroides*, a species that has become enormously problematic in recent decades. More generally, this suggests that species that are adapted to stressful environments, may be more likely to evolve herbicide resistance (Matzrafi et al., 2016a).

Our results confirm that there is a relationship between the mechanisms that endow resistance in weeds in general and resistance to abiotic stress. While the mechanisms that typically govern NTSR in weeds are a subset of the mechanisms that govern physiological responses to abiotic stresses, it has also been emphasized that under different environmental conditions, factors such as herbicide modes of action and the physiology of the weed species can participate significantly in the alteration of NTSR (Cummins et al., 1997; Jugulam & Shyam, 2019; Letouze & Gasquez, 2001; Rosenhauer et al., 2015). Also, it has been shown that ambient conditions, such as temperature, can affect responses of weeds to herbicides (Vila‐Aiub et al., 2013). Interactions between stress tolerances have been noted previously. For example, work on *Poa pratensis* established that prior exposure to freezing significantly impacts survival and growth following subsequent exposure to drought (Kong & Henry, 2016). However, to our knowledge, this study is the first to demonstrate that exposure to drought stress may directly confer herbicide resistance in subsequent generations.

### 4.1 The evolution of herbicide resistance in weeds through physiological pathways

A major agronomic threat from NTSR is that this form of resistance confers resistance across herbicides. Previously it has been reported that occurrence of metabolic “cross‐resistance” in *Lolium rigidum* to different herbicides may be either via the P450 (Morant et al., 2003) or other metabolism genes (e.g., GT and GST) (Yu & Powles, 2014). These can metabolize a number of herbicides, thus resistance arises (Busi & Powles, 2013; Yu & Powles, 2014). Our results support the suggestion that environmental conditions may also play a role in metabolic resistance evolution, as the enzymes involved (e.g., P450s and GSTs) can mediate respond to biotic or abiotic stresses (Marrs, 1996; Schuler & Werck‐Reichhart, 2003; Yu & Powles, 2014). Plant GSTs bind glutathione to electrophilic xenobiotics, which marks them for sequestration with vacuolar impact. The role of GSTs in metabolism is uncertain, nonetheless their complicated environmental stimulus management suggests that they have vital defensive functions (Edwards et al., 2000). In normal plant growth and plant stress responses, the plant GSTs perform a number of key catalytic and non‐enzymatic functions (Dixon et al., 2002).

Reade et al. (2004) concluded that GSTs may defend against herbicides when their activity or abundance increases, even if they are not contributing directly to herbicide metabolism. It has been confirmed that the contribution of GSTs in the evolution of multiple herbicide resistance (MHR) in *A*. *myosuroides* occurs through oxidative stress tolerance as well as detoxifying herbicides by stimulating their conjugation with glutathione (Cummins et al., 1999; Preston et al., 1996). Thus, this mechanism is presumed to be responsible for the evolution of herbicide resistance in grass weed populations that have been exposed to abiotic stress, such as drought.

That such routes are involved in herbicide resistance is supported by research on the impact on plant transcriptome or proteome of herbicide applications, which indicate that response to herbicide stress can be correlated with response to other stresses (Das et al., 2010; Unver et al., 2010; Vivancos et al., 2011). Environmental conditions have a major effect on the evolution of resistance to different herbicide through metabolic detoxification mechanism, such as temperature (Ge et al., 2011; Matzrafi et al., 2016b; Yu et al., 2009). The results of the current study indicate that drought stress can affect the efficiency of herbicide to control the weed species. For example, the result of survival and dry weight of offspring both in resistant and damaged plants presented here suggests that exposure to high drought stress can result in failed weed treatment. These results underline the importance of environmental conditions after application of herbicide (Matzrafi et al., 2016b).

### 4.2 The possible roles of epigenetic mechanisms in herbicide resistance

We wished to establish whether evolution of herbicide resistance might be underpinned via epigenetic mechanisms. Our study provides clear evidence that exposure of grass weed *A*. *myosuroides* populations to drought stress can confer herbicide resistance in subsequent generations, and that the mechanism conferring heritability of herbicide resistance is non‐genetic.

Plants that previously experienced a type of stress may change plants following responses toward next stress by producing more rapid and/or stronger reactions which mean plants exercise a form of ‘stress memory’ (Ding et al., 2012; Miryeganeh, 2021; Walter et al., 2013). In addition, evolved tolerance for abiotic stress after previous exposure to stress has been called the ‘priming effect’ (Tanou et al., 2012), and has been reported for drought, pathogens, inundation, and fire in previous studies (Li et al., 2011; Onate et al., 2011). This is a phenomenon known as plant hardening (Boyko & Kovalchuk, 2011), by which stress can act as a signal for future more severe stress, which can stimulate mechanisms producing superior stress tolerance (Beck et al., 2004).

Herbicide resistance is typically thought to evolve through the action of natural selection on standing genetic variation within plant populations (Neve & Powles, 2005). Until now, the roles of few non‐genetic factors in the evolution of herbicide resistance have been investigated (Delye et al., 2013). Epigenetic mechanisms may mean that the environment can affect gene expression without DNA sequences (Concenço, 2016). Non‐genetic processes are widely involved in the regulation of stress responses (Boyko & Kovalchuk, 2008), and gene silencing is one of the epigenetic mechanisms of most concern for herbicide resistance in plants (Concenço, 2016). Epigenetic mechanisms have been highlighted an important mediators of interactions between plants and their response to the environment, largely linked with stress adaptation (Markus et al., 2018; Miryeganeh, 2021).

Our results for the first time clearly provide evidence that offspring of *A*. *myosuroides* plants exposed to high drought stress acquire stronger defense mechanism to resist herbicide. This mechanism may explain why the grass weed *A*. *myosuroides* so readily evolves mechanisms to inhibit or minimize damage or mortality through resistance. However, more researches required to investigate whether the resistance will occur in the following generations and does the populations response regarding the resistance will be the same to different herbicides.

## CONFLICTS OF INTEREST

The authors declare that they have no conflicts of interest.

## AUTHOR CONTRIBUTION

**Vian H. Mohammad:** Conceptualization (equal). **Colin P. Osborne:** Conceptualization (equal). **Robert P. Freckleton:** Conceptualization (equal).

## AUTHORS’ CONTRIBUTIONS

The study was conceived by VM, RF, and CO. VM undertook the experiments. The data were analyzed by VM and RF. All authors were involved in the writing of the manuscript.

## Supporting information

Figure S1‐S2

Table S1‐S8

## Data Availability

All data will be archived on figshare (https://figshare.shef.ac.uk/).
