# Supplementary material for: Drought exposure leads to rapid acquisition and inheritance of herbicide resistance in the weed Alopecurus myosuroides
Source: Ecol Evol. 2022 Feb 16;12(2):e8563. doi: 10.1002/ece3.8563 (PMC8848470; doi:10.1002/ece3.8563)
Supplement: Supplementary file 1 — Figure S1‐S2 [file ECE3-12-e8563-s001.docx]

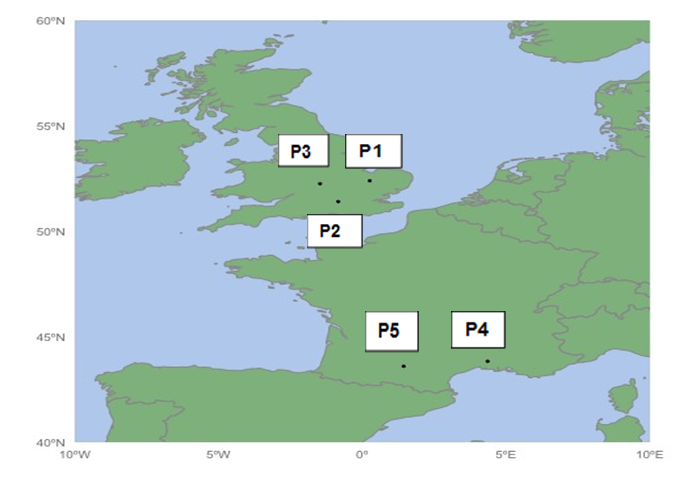


**Figure S1.** Shows the locations of sampling sites of the A. myosuroides populations that used in the first two experiments. Black dots on the map represent the locations of the populations (P). P1 = Ely Cambridgeshire, UK; P2 = Wokingham Berkshire, UK; P3 = Warwickshire, UK; P4 = Nimes Languedoc-Roussillon, France; P5 = Toulouse Pyrenees, France.


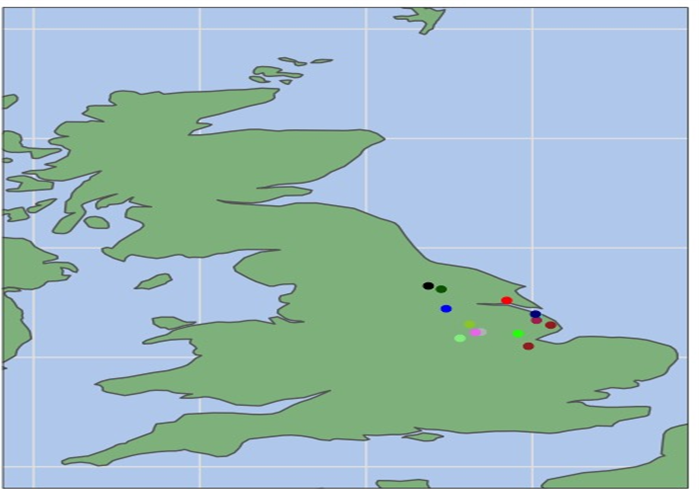


**Figure S2.** The dots on the map represent the locations of sampling the Alopecurus myosuroides weed. Each dot represent a population "P = Population" and each population is indicated by a different colour. P1 = Black; P2 = Red; P3 = Gray; P4 = Blue; P5 = Green; P6= Turquoise; P7= Violet; P8= Maroon; P9= Dark-orange; P10= Dark-red; P11= Yellow-green; P12= Orange; P13= Brown; P14= Dark-green and P15= Dark-blue.
