# Supplementary material for: Drought exposure leads to rapid acquisition and inheritance of herbicide resistance in the weed Alopecurus myosuroides
Source: Ecol Evol. 2022 Feb 16;12(2):e8563. doi: 10.1002/ece3.8563 (PMC8848470; doi:10.1002/ece3.8563)
Supplement: Supplementary file 2 — Table S1‐S8 [file ECE3-12-e8563-s002.docx]

**Table S1.** Plant height, biomass and seed weight per plant of A. myosuroides under two different levels of drought stress medium and high (75% and 25% plant mortality). All the values are the logarithm of parameters value. Mean ± standard error are shown for A. myosuroides plants across all the populations, where the drought effect was significant. Asterisks indicate significant differences for both drought stress levels in comparison with controls. Significance levels from the ANOVAs are shown in the following way: p< 0.05, *, p< 0.01, **, p<0.001, ***.

|  | Degrees of freedom | Sum of Squares | Mean Square | F-value | *p*-value |
| --- | --- | --- | --- | --- | --- |
| *Alopecurus myosuroides* |  |  |  |  |  |
| log (plant height) |  |  |  |  |  |
| Replicate | 3 | 0.297 | 0.099 | 1.797 | 0.160 |
| Population | 4 | 1.068 | 0.267 | 4.847 | 0.002** |
| Treatment | 2 | 1.630 | 0.815 | 14.794 | < 0.0001*** |
| Residuals | 50 | 2.755 | 0.055 | - | - |
| log (biomass) |  |  |  |  |  |
| Replicate | 3 | 0.465 | 0.155 | 2.168 | 0.104 |
| Population | 4 | 0.206 | 0.052 | 0.723 | 0.581 |
| Treatment | 2 | 6.022 | 3.011 | 42.135 | < 0.0001*** |
| Residuals | 50 | 3.573 | 0.072 | - | - |
| log (seed weight) |  |  |  |  |  |
| Replicate | 3 | 0.446 | 0.149 | 0.286 | 0.835 |
| Population | 4 | 9.290 | 2.323 | 4.473 | 0.004** |
| Treatment | 2 | 35.179 | 17.590 | 33.877 | < 0.0001*** |
| Residuals | 49 | 25.441 | 0.519 | - | - |

**Table S2.** **a.** Analysis of F1 generation plants that are resistant + damaged (i.e. survived) versus dead plants for A. myosuroides. Results are from generalized linear models with binomial error and a log-link function. **b.** Analysis of F1 generation plants that are resistant (i.e. survived intact) versus dead or damaged for A. myosuroides. Results are from generalized linear models with binomial error and a log-link function. Significance of model terms indicated by asterisks: *, p < 0.05 and, **, p < 0.01. Significance of model terms indicated by asterisks: *, p< 0.05 and, ***, p< 0.001.

**a.**

|  | d.f. | Deviance | Residual d.f. | Residual Deviance | *p*-value |
| --- | --- | --- | --- | --- | --- |
| Null |  |  | 129 | 172.260 |  |
| Blocks | 4 | 4.2517 | 125 | 168.008 |  |
| Population | 4 | 9.6903 | 121 | 158.318 | * |
| Herbicide | 1 | 31.2973 | 120 | 127.020 | *** |
| Drought | 2 | 7.8589 | 118 | 119.162 | * |
| Herbicide*drought | 2 | 28.3554 | 116 | 90.806 | *** |

|  | d.f. | Deviance | Residual d.f. | Residual Deviance | *P*-value |
| --- | --- | --- | --- | --- | --- |
| Null |  |  | 129 | 172.283 |  |
| Blocks | 4 | 6.0950 | 125 | 121.188 |  |
| Population | 4 | 13.0558 | 121 | 108.133 | * |
| Herbicide | 1 | 1.0195 | 120 | 107.113 |  |
| Drought | 2 | 13.0938 | 118 | 94.019 | ** |
| Herbicide*drought | 2 | 1.2682 | 116 | 94.751 |  |

**b.**

**Table S3.** Results from analysis of variance of linear models describing the relationship between variance, drought, herbicide and drought*herbicide interaction for dry weight of surviving plants for A. myosuroides. Significance of model terms indicated by asterisks: **, p< 0.01 and ***, p< 0.001.

|  | Degrees of freedom | Sum of Squares | Mean Square | F-value | *p*-value |
| --- | --- | --- | --- | --- | --- |
| Blocks | 4 | 9.0963 | 2.2741 | 8.2263 | <0.001*** |
| Population | 4 | 1.1589 | 0.2897 | 1.0480 | 0.3892 |
| Drought | 2 | 0.5039 | 0.2519 | 0.9113 | 0.4069 |
| Herbicide | 1 | 0.0068 | 0.0068 | 0.0244 | 0.8762 |
| Drought*herbicide | 2 | 2.8734 | 1.4367 | 5.1972 | 0.0080** |
| Residuals | 67 | 18.5216 | 0.2764 | - | - |

**Table S4.** Results of Linear Mixed Models fit by REML; t-test use Satterthwaite's method describe the effect of high drought stress treatment on growth of cloned parental generation of 15 populations of A. myosuroides. The model included drought treatment as a fixed effects and clone.ID as a random effects. Data on (A) plant height, (B) biomass and (C) seed weight were log transformed. Significance of model terms indicated by asterisks: *** p< 0.001.

| Fixed effects | Estimate | | Std. Error | t value | Satterthwaite *p* |
| --- | --- | --- | --- | --- | --- |
| A) Plant height |  | |  |  |  |
| drought | -0.47 | | 0.06 | -7.67 | 6.08 x 10^-12^ *** |
| B) Plant biomass | |  |  |  |  |
| drought | -0.70 | | 0.10 | -7.29 | 4.16 x 10^-11^ *** |
| C) Seed weight |  | |  |  |  |
| drought | -1.55 | | 0.21 | -7.48 | 7.22 x 10^-11^ *** |

**Table S5.** Results of generalized linear mixed model fit by maximum likelihood (Laplace approximation) with binomial error (logit), for the effect of drought stress treatment on survivorship of cloned parental generation of 15 populations of A. myosuroides. The first column: Estimate, refers to the estimated value of the beta coefficient. The second column, Std. Error, is the standard error of the estimate betas. The last two columns characterise z-values of the estimated beta coefficients and the p-value. Significance of model terms indicated by asterisks: *** p< 0.001.

| Fixed effects | Estimate | Std. Error | z value | Pr (>\|z\|) |
| --- | --- | --- | --- | --- |
| intercept | 1.70 | 0.22 | 7.61 | 2.74 x 10^-14^ *** |
| drought | -2.27 | 0.28 | -8.02 | 1.04 x 10^-15^ *** |

**Table S6.** Reports the results of generalized linear mixed model fit by maximum likelihood (Laplace approximation) with binomial error (logit), for F1e generation plants that are (A) resistant vs damaged + dead plants, and (B) resistant + damaged vs dead plants of A. myosuroides. The first column: Estimate, refers to the estimated value of the beta coefficient. The second column, Std. Error, is the standard error of the estimate betas. The last two columns characterise z-values of the estimated beta coefficients and the p-value. Significance of model terms indicated by asterisks: ** p < 0.01, *** p < 0.001.

| Fixed effects | Estimate | Std. Error | z-value | Pr (>\|z\|) |
| --- | --- | --- | --- | --- |
| A) resistant vs (damaged + dead) plants |  |  |  |  |
| Intercept | -1.376 | 0.528 | -2.607 | 0.009 ** |
| herbicide-sublethal dose | 1.592 | 0.291 | 5.476 | 4.36 x 10^-8^ *** |
| drought | 3.669 | 0.448 | 8.188 | 2.67 x 10^-16^ *** |
| herbicide-sublethal dose: drought | -1.863 | 0.580 | -3.211 | 0.0013 *** |
| B) (resistant + damaged) vs dead plants |  |  |  |  |
| Intercept | 2.10 | 0.76 | 2.72 | 0.007 ** |
| herbicide-sublethal dose | 2.12 | 0.48 | 4.41 | 1.04 x 10^-5^ *** |
| drought | 3.47 | 0.84 | 4.16 | 3.21 x 10^-5^ *** |
| herbicide-sublethal dose: drought | -3.67 | 1.15 | -3.20 | 0.001 ** |

**Table S7.** Results of ANOVA from Linear Model describing the effect of drought stress in cloned parental generation on the response of F1e resistant dry weight after fenoxaprop-p-ethyl herbicide application (lethal and sublethal doses). Data on dryweight of resistant plants were log transformed. Significance of model terms indicated by asterisks: *** p < 0.001.

|  | Degrees of freedom | Sum of squares | Mean of squares | F-values | *p*-value |
| --- | --- | --- | --- | --- | --- |
| log (resistant. Dryweight) |  |  |  |  |  |
| Clone.ID | 4 | 5.03 | 1.26 | 1.39 | 0.24 |
| Drought | 1 | 73.57 | 73.57 | 81.27 | 1.14 x 10^-14^ *** |
| Herbicide | 1 | 0.28 | 0.28 | 0.30 | 0.58 |
| Residuals | 103 | 93.24 | 0.91 |  |  |

**Table S8.** Results of Linear Mixed Models fit by REML; t-tests use Satterthwaite's method describe the effect of High drought stress treatment on cloned parental generation on the response of F1e damaged dry weight after fenoxaprop-p-ethyl herbicide application (lethal and sublethal doses). Data on dryweight of damaged plants were log transformed. The model included drought and herbicide treatment as a fixed effects and clone.ID as a random effects. Significance of model terms indicated by asterisks: ** p < 0.01

|  | Estimate | Std. Error | t value | Pr(>\|t\|) |
| --- | --- | --- | --- | --- |
| log(dryweight of damaged plants) |  |  |  |  |
| Herbicide lethal dose | -0.19 | 0.26 | -0.74 | 0.489 |
| Herbicide sublethal dose | -0.05 | 0.28 | -0.19 | 0.852 |
| drought | 1.36 | 0.39 | 3.46 | 0.001 ** |
